# B-cell subsets in leprosy lesions: unraveling the complex interplay^؆^

**DOI:** 10.1016/j.abd.2025.501184

**Published:** 2025-08-09

**Authors:** Luis Alberto Ribeiro Fróes, Carla Pagliari, Maria Angela Bianconcini Trindade, Mirian Nacagami Sotto

**Affiliations:** a Department of Pathology, Faculty of Medicine, Universidade de São Paulo, São Paulo, SP, Brazil; b LIM56, Hospital das Clínicas, Faculty of Medicine, Universidade de São Paulo, São Paulo, SP, Brazil; c Instituto de Medicina Tropical da Faculty of Medicine, Universidade de São Paulo, São Paulo, SP, Brazil

**Keywords:** B-lymphocyte subsets, Immune response, Immunohistochemistry, Leprosy

## Abstract

**Background:**

Leprosy is a chronic infectious disease marked by complex immune interactions, yet the roles of specific B-lymphocyte subsets in its pathology are poorly understood.

**Objectives:**

To investigate the presence and distribution of B-cell subsets, including B1 cells, Marginal Zone (MZ) B-cells, Regulatory B-cells (Bregs), and Effector-1 B-cells (Be1), across different clinical forms of leprosy and reactional states.

**Methods:**

Immunohistochemical and morphometric analyses were performed on skin lesions from patients with various clinical presentations of leprosy.

**Results:**

CD20+ B-cells were abundant in tuberculoid lesions, whereas MZB-1 expression varied significantly among leprosy subtypes. Type 1 Reaction (T1R) lesions exhibited significantly higher counts of B1 and MZ B-cells compared to Type 2 Reaction (T2R), Lepromatous Leprosy (LL), and Indeterminate leprosy (I). Expression patterns of PAX5/MZB-1 and PAX5/CD5 suggested a dominant presence of these cells in the Th1 pole. Be1 cells, strongly linked to Th1 immune response, were also more abundant in Th1 clinical presentations (tuberculoid and T1R leprosy). Although Bregs were generally scarce, they were most frequently observed in T1R.

**Study limitations:**

This study was limited by the relatively small number of cases analyzed per clinical subtype and reactional state.

**Conclusions:**

This is the first study to document the presence and distribution of these specific B-cell subsets in leprosy lesions. The findings suggest distinct roles for B-lymphocyte subtypes, particularly at the tuberculoid pole and during Type 1 reactions.

## Introduction

The spectral manifestations of leprosy reflect the host's immune response against *Mycobacterium leprae*.^1^ As an obligate intracellular bacterium, the effectiveness of the immune response against leprosy primarily relies on cellular immunity, with the balance between Th1 and Th2 playing a pivotal role in determining the clinical presentation of the disease. The progression from tuberculoid (localized) to lepromatous (disseminated) leprosy is marked by a shift from Th1 (cellular) to Th2 (humoral) response. Consequently, the cytokine profile varies between presentations, with Th1 cytokines (such as IFN-γ, IL-2, and IL-12) in tuberculoid leprosy and Th2 cytokines (such as IL-4, IL-5, IL-10, and TGF-β) in lepromatous leprosy.^2^

“Indeterminate” leprosy presents with a hypochromic macule with diminished local sensitivity, potentially evolving with spontaneous healing or progressing to a classic presentation. This type of leprosy has no clear predominance of Th1 or Th2 response.^1^

Leprosy reactions are episodes of acute hypersensitivity characterized by the worsening of previous lesions or the appearance of new ones. Type 1 reaction is a type IV hypersensitivity reaction characterized by an exacerbation of the cellular immune response against *M. leprae*, with significant secretion of TNF-α, IFN-γ, IL-2, IL-1β, and IL-6.^3^ Type 2 reaction involves a systemic inflammatory process related to extravascular deposition of immune complexes and neutrophilic exudate, with high levels of TNF-α, IL-2, IL-4, IL-5, IL-6, and IL-10.^3^

In 1993, Modlin et al.^4^ published studies linking the clinical spectrum of leprosy to Th1-Th2 responses. Since then, most of the research in leprosy immunology has concentrated on T-cells. Although Ridley et al. first described the presence of B-cells in 1974, the focus on T-cells over the decades led to limited investigation into the role of B-cells.^5^ It was only recently, in 2017, that Fachin et al.^6^ revealed an increased presence of B-cells in the Th1 pole, challenging the previous assumption that B-cells would be predominantly associated with the Th2 pole, which is typically linked to humoral responses.

Mammalian B-cells encompass distinct subsets, including Follicular B2 cells, B1-cells, Marginal Zone B-cells (MZB), B-Regulatory cells, and B-Effector-1 (Be1) cells.^7^, ^8^ B1-cells, originating during fetal development, inhabit the peritoneal cavity, exhibiting self-renewal and spontaneous IgM secretion. Despite controversies in the literature regarding the characterization of B1 B-cells in humans,^9^, ^10^, ^11^ reports indicate that they constitutively express CD5 (B1a cells) and spontaneously secrete multi-reactive IgM, facilitating early bacterial clearance.^12^

MZ B-cells, another category of B-lymphocytes, inhabit the marginal zone of lymphoid follicles within the spleen and several other tissues.^13^ These cells orchestrate swift antibody responses against blood-borne microbes rich in polysaccharides. Along with B1-cells, a subset of MZ B-cells, specifically the transitional type 2 marginal zone precursor (T2-MZP) B-cells, are termed Innate-Like B-Lymphocytes (ILBs) or innate B regulatory cells (Bregs) due to their ability to secrete IL-10, a regulatory cytokine.^14^ Bregs contribute to cutaneous immune responses and exhibit both pro-inflammatory and regulatory actions.^15^

A recently recognized B-cell subset, the B-effector-1 (Be1) cell, shares resemblances with T-helper-1 (Th1) cells and seems to bridge innate and adaptive immune responses.^16^, ^17^ Be1 cells release inflammatory Th1-like cytokines such as IFN-γ, TNF-α, and IL-12.^18^, ^19^

By dissecting the intricate interplay between these B-cell subsets and the mycobacterial environment within leprosy lesions, the present investigation aims to provide a comprehensive understanding of the in situ immune response in leprosy. The present findings hold the potential to inform therapeutic strategies and refine disease management approaches, opening new avenues for effective intervention.

## Materials and methods

This study involves a case series examination. Initially gathered for diagnosing and classifying classical forms of leprosy and reactive states, skin samples have been selected from the archives of the Dermatopathology Laboratory at the Dermatological Clinic, Department/Division of the University of São Paulo Medical School. These samples were processed using histological techniques and embedded in paraffin. The authors examined skin samples from 95 patients with various clinicopathological manifestations of leprosy. These included Lepromatous leprosy (18 samples), Tuberculoid leprosy (23 samples), Indeterminate leprosy (14 samples), Type 1 Reaction (19 samples), and Type 2 Reaction (21 samples). Specifically, for comparisons within the CD20 group, the authors utilized a control group comprising samples of normal skin from 10 patients. In contrast to Dendritic and T-cells, B-cells are known to be very scarce in normal skin under homeostatic conditions.^20^ Nonetheless, the authors established a dedicated control group for CD20 to reaffirm this observation and eliminate the need for controls in B-cell subsets. All histological samples of leprosy lesions were stained with hematoxylin-eosin and the acid-fast bacilli staining technique (Faraco technique) for diagnostic confirmation. Additionally, patient records were consulted to aid in the classification and composition of study groups.

### Immunohistochemical techniques

Four micrometer paraffin-embedded sections were obtained from all specimens and subjected to single-labeling techniques using CD20 (B-cells) and MZB-1 (B1-cells, MZB-cells, and plasmablasts/plasma cells) antibodies.^20^ For better discrimination between the MZB-cell population and plasmablasts/plasma cells at the lesion sites, the authors performed double labeling PAX5/MZB-1 to distinguish the MZB/B1-cells in the tissue from plasmablasts and plasma cells, which have silenced PAX5 expression.^21^ The authors also performed double-staining for PAX5/CD5 (B1-cells), CD20/T-bet (Be1-cells), and CD20/c-MAF (Bregs) with a protocol modified by Tudor et al.^22^ For Bregs, the authors initially attempted to perform dual labeling with PAX5/IL-10; however, due to unsatisfactory results, the authors opted for labeling surface CD20 with the IL-10 nuclear transcription factor, c-MAF, also expressed by regulatory B-lymphocytes.^23^, ^24^, ^25^ The choice for CD20/c-MAF yielded more easily discernible and reliable results. Table 1 displays the antibody specifications, detection systems, and chromogens used for the immunohistochemical procedures. Detailed technique descriptions are given as Supplementary data.

**Table 1:** Antibodies, detection systems, and chromogens specifications.

Primary antibody	ID Code	Manufacturer	Dilution/Detection system
Anti-CD5 rabbit antibody	SP-19	Cell-marque	1:50/Polink
Anti-PAX5 mouse antibody	312M-15	Cell-marque	1:200/Envision flex
Anti-CD20 mouse antibody	AC-0012	Epitomics	1:100/Envision flex
Anti-MZB-1 rabbit antibody	11454-1	Proteintech	1:200/Polink
Anti-Tbet rabbit antibody	aB-150440	ABcam	1:200/Polink
Anti-c-MAF rabbit antibody	SC7866	Vector	1:100/Impress

Chromogens used for double labeling technique	ID Code	Manufacturer	Dilution/Detection system
Membrane/cytoplasm			
PermaGreen / HRP substrate	K074-RUO	Diagnostic BioSystems	Not applicable
Nucleus			
3.3′-Diaminobenzidine tetrahydrochloridrate	D5637-50G	Sigma Life Science	Not applicable

### Fluorescence confocal microscopy

The immunofluorescence technique protocol for confocal microscopy was performed as follows: both primary antibodies, anti-IgM, and anti-PAX5, were diluted and mixed in 1% bovine albumin solution and incubated for 48 hours at 4 °C. Following, it was applied the isotype-specific secondary conjugated antibodies Alexa Fluor® 488 (green) goat anti-rabbit Ig and AlexaFluor® 555 (red) donkey anti-mouse Ig, Invitrogen, Carlsbad, CA, USA, and nuclear staining with 6-Diamidino-2-Phenylindole, Dihydrochloride DAPI - Sigma-Aldrich, Steinhein, Germany (blue) for 60-minutes. The slides were mounted in a mounting medium (Hydromount, National Diagnostics, Atlanta, GA, USA) and glass coverslips and maintained protected from light at 4 °C until analysis. Fluorescent images were acquired using UV/Laser excitation on confocal microscopy Olympus FluoView 1000 (FV1000). Given the limited number of cases analyzed, a total of 5, quantitative morphometric analyses were not conducted.

### Morphometric and statistical analyses

All slides immunostained with CD20 and MZB-1 antibodies were scanned with the 3DHISTECH “whole slide imaging” system (Budapest, Hungary). For morphometric analysis, images with a digital zoom of 10× were obtained from successive, non-superimposable fields of the entire dermis for each sample, using Case Viewer software (2.3 version 64-bit, 3DHISTECH Ltd.).

The images were processed using the freely available Fiji/ImageJ software from the National Institutes of Health (http://imagej.nih.gov). Following the methodological approach outlined by Biswas et al.,^25^ the authors comprehensively analyzed all images associated with each sample. The arithmetic means of the immunostained area fraction, specifically for CD20 and MZB-1, was then calculated for each sample (case). In analyses involving colocalization of antigens, double positive cells were manually counted in each *hotspot* of whole-slide images, divided by the total number of B-cells in the same field, and then by the field dermal area (estimated by the free Fiji/ImageJ software). After analyzing all images for each sample, an arithmetic mean was calculated for the proportion of double immunostained cells to the total B-cells (CD20 or PAX5 positive cells) per unit of area. Thus, the use of the term 'expression' later in the text refers specifically to the number of positive cells or the fraction of immunolabeled tissue, rather than to staining intensity.

Non-parametric one-way ANOVA followed by Kruskall Wallis' multiple comparisons test was performed using GraphPad Prism version 10.1.0 for macOS, GraphPad Software, Boston, Massachusetts USA, www.graphpad.com. A p-value < 0.05 was considered statistically significant.

## Results

Samples from 95 cases and 10 controls were analyzed. Among the cases, the male-to-female ratio was 11:9, indicating a slightly higher representation of males. This group had an average age of 44.64, from 8 to 84 years. In the control group, the average age was (44.7-years.) with a corresponding male-to-female ratio of (5:5).

### Presence and distribution of single-labeled cells

**Fig. 1:**
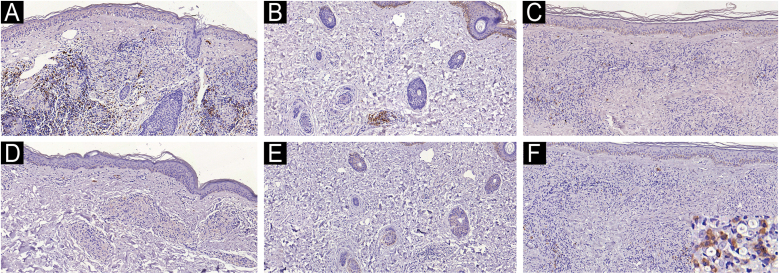
B (CD20+) cells in tuberculoid leprosy arranged around granulomas (A) and cells immunolabeled with the MZB1 antibody (D). Presence of rare perivascular B-cells (B) and absence of MZB1 expression (E) in indeterminate leprosy. In lepromatous leprosy, note rare clusters of B-cells within the diffuse macrophage infiltrate (C) and rare cells immunolabeled with the MZB1 antibody, some of them with plasma cell morphology (inset) (F). Immunohistochemical technique, diaminobenzidine chromogen, and counterstaining with hematoxylin. Scale bars (left top) indicate the magnification.

**Fig. 2:**
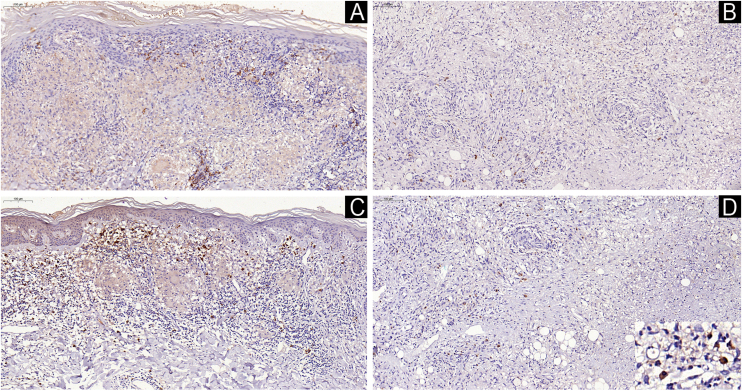
In type 1 reaction, B (CD20+) cells are present in the papillary dermis and around epithelioid granuloma in the reticular dermis (A). Cells immunolabeled with the MZB1 antibody are observed in the papillary dermis; however, they are absent around the granuloma (C). In type 2 reaction, rare B-cells amidst the regressive macrophage infiltrate (B) and a few clusters of cells immunolabeled with the MZB1 antibody, some of them with plasma cell morphology (inset) (D). Immunohistochemical technique, diaminobenzidine chromogen, and counterstaining with hematoxylin. Scale bars (left top) indicate the magnification.

The presence and distribution of B-lymphocytes differed in the tissue response among the studied leprosy groups. B-cells were observed in the papillary dermis and around epithelioid granulomas in tuberculoid leprosy samples (Fig. 1A). In indeterminate leprosy, B-cells were scarce and, when present, were located near blood vessels and cutaneous adnexa (Fig. 1B). Rare lymphocytes were observed alongside vacuolated macrophages in the tissue response of lepromatous leprosy samples (Fig. 1C). B-cells were slightly more abundant, arranged around granulomas, in type 1 reaction leprosy (Fig. 2A) and scarce in type 2 reaction leprosy skin samples (Fig. 2B).

Cells immunostained with the MZB-1 antibody exhibited morphology, sometimes like lymphocytes, sometimes slightly larger and resembling plasma cells. They appeared scarcer than CD20 in tuberculoid leprosy samples (Fig. 1D) and extremely scarce or absent in indeterminate leprosy samples (Fig. 1E). On the other hand, in lepromatous leprosy samples, MZB-1 positive cells were more frequently observed than B-cells and exhibited plasma cell and plasmablast morphology (Fig. 1F). In type 1 reaction samples MZB-1-positive cells were distributed in the papillary dermis and, in some cases, they were scarce or even absent around granulomas (Fig. 2C) contrary to what was observed in tuberculoid leprosy samples. Type 2 reaction samples displayed MZB-1-positive cells forming small clusters, and like lepromatous leprosy samples, they showed a morphological appearance of plasma cells (Fig. 2D).

Given that the MZB-1 antibody reveals the MZB-1 protein, associated with the processing and secretion of immunoglobulins and present in the endoplasmic reticulum of B-cells in the marginal zone of lymphoid follicles, plasmablasts, and plasma cells,^20^ the authors decided to compare the mean expression area fraction of CD20 (B-cell marker) with the mean expression area fraction of MZB-1 in each leprosy group. That was an attempt to ascertain whether MZB-1 expression was solely attributable to the presence of plasmablasts and plasma cells in the tissue response of the studied samples. Table 1S in Supplementary materials demonstrates the statistical analysis results of these comparisons.

### Presence and distribution of double-labeled cells

**Fig. 3:**
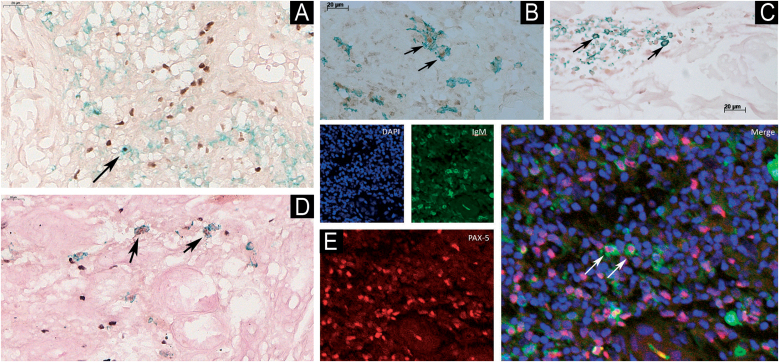
B1-cells (PAX5+/CD5+) in tuberculoid leprosy (A); Be1-cell (CD20+/Tbet+) in type 1 reaction (B); Breg cell (CD20+/c-MAF+) in type 1 reaction (C); B1 and MZB cells (PAX5+/MZB1+) in tuberculoid leprosy (D), double immunolabeling technique using diaminobenzidine chromogen (brown) for PAX5 and T-bet and PermaGreen (green) for CD20 antibody; B1-cell (PAX5+/IgM+) in laser confocal microscopy in type 1 reaction (E). Arrows indicate the respective cells. Scale bars – indicate the magnification.

The authors observed a sparse presence of double-labeled cells in all evaluated B-cell subpopulations. Fig. 3 (A, B, C, and D) illustrates some of these cases, contrasting with the infiltrates shown previously for CD20 mainly in tuberculoid and type 1 leprosy reaction samples. In addition, slide reading using the Confocal Immunofluorescence Microscopy technique revealed the presence of PAX5/IgM (B1-cells) in cases of T1R (Fig. 3E).

### Morphometric and statistical analyses

**Fig. 4:**
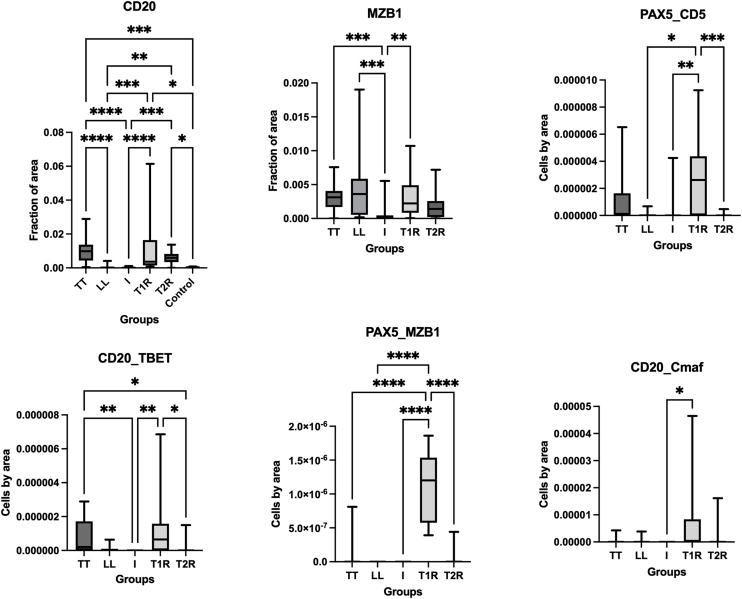
Medians for all groups in graphical form, along with their corresponding measures of central dispersion. The asterisks indicate statistically significant post hoc comparisons. LT, Tuberculoid Leprosy; I, Indeterminate leprosy; LL, Lepromatous Leprosy; T1R, Type 1 leprosy Reaction; T2R, Type 2 leprosy Reaction.

The mean rank differences observed in Dunn's multiple comparisons test delineate distinctions in the ratio of double-labeled positive cells in comparison to single-positive PAX5 or CD20 cells per unit area among distinct leprosy groups. The differences in medians between groups with statistically significant comparisons are summarized in Table 2, along with their respective p-values. Fig. 4 displays the medians for all groups in graphical form, along with their corresponding measures of central dispersion.

**Table 2:** Differences in medians between the groups with statistically significant comparisons and their respective p-values.

CD20		
Dunn's multiple comparisons test	Mean rank difference	Adjusted p-value
LT vs. LL	51.82	<0.0001
LT vs. I	58.67	<0.0001
LT vs. Controls	48.57	0.0002
LL vs. T1R	-41.31	0.0004
LL vs. T2R	-37.8	0.0016
I vs. T1R	-48.17	<0.0001
I vs. T2R	-44.66	0.0002
T1R vs. Controls	38.07	0.0145
T2R vs. Controls	34.56	0.0374

MZB-1		
Dunn's multiple comparisons test	Mean rank difference	Adjusted p-value
TT vs. I	35.07	0.0006
LL vs. I	36.58	0.0007
I vs. T1R	-32.17	0.0066

PAX5/CD5		
Dunn's multiple comparisons test	Mean rank difference	Adjusted p-value
LL vs. T1R	-22.93	0.0245
I vs. T1R	-30.86	0.0024
T1R vs. T2R	30.17	0.0005

CD20/Tbet		
Dunn's multiple comparisons test	Mean rank difference	Adjusted p-value
TT vs. I	27.33	0.0055
TT vs. T2R	22.86	0.0123
I vs. T1R	-27.29	0.0096
T1R vs. T2R	22.82	0.0227

CD20/c-Maf		
Dunn's multiple comparisons test	Mean rank difference	Adjusted p-value
I vs. T1R	-15.74	0.0373

PAX5/MZB-1		
Dunn's multiple comparisons test	Mean rank difference	Adjusted p-value
TT vs. T1R	-31.18	<0.0001
LL vs. T1R	-37.5	<0.0001
I vs. T1R	-37.5	<0.0001
T1R vs. T2R	35.53	<0.0001

LT, Tuberculoid form; I, Indeterminate form; LL, Lepromatous form; T2R, Type 2 leprosy Reaction; T1R, Type 1 leprosy Reaction.

## Discussion

The present study observed a higher area fraction of CD20+ cells (B-cells) in the tuberculoid pole of leprosy compared to the lepromatous pole, which is consistent with recent literature findings.^25^, ^26^ The lepromatous leprosy group, however, exhibited a lower B-cell component compared to the reactional groups, both Type 1 (T1R) and Type 2 (T2R).

The involvement of B-cells in mycobacterial infections has been discussed in the literature, particularly in the context of granuloma formation, cytokine production, T-cell responses, and antigen presentation.^27^ This could explain the higher expression of CD20 and the presence of B-cells in the granulomatous response group, aligning with literature data. Interestingly, this study demonstrated that reactional states (T1R and T2R) exhibited a higher population of B-cells compared to the lepromatous group. In T1R, this could be related to the better organization of the granulomatous response. In T2R, however, these findings differ from those reported by Biswas et al.,^25^ who found fewer B-lymphocytes in T2R compared to multibacillary leprosy. These authors correlated the presence of B-lymphocytes in T2R with poorly formed granulomas, which might not fully align with the present observations.

Lesions from indeterminate leprosy demonstrated lower B-cell expression compared to the tuberculoid group and the reactional groups (T1R and T2R). This might be due to the less intense inflammatory response or the undefined polarization of this group.

The MZB-1 molecule, encoded by the MZB-1 gene, is present in the endoplasmic reticulum and functions as a chaperone involved in the folding and secretion of Immunoglobulin M (IgM). MZB-1 is a marker for B-lymphocytes, particularly B1-cells, Marginal Zone-B (MZB) cells, plasmablasts, and plasma cells.^28^ B1-cells are known for secreting IgM, characterizing them as part of the early humoral immune response. The authors chose this marker to demonstrate the presence or absence of this B-lymphocyte subtype in leprosy lesions. However, the authors could demonstrate the expression of IgM in PAX5-positive cells (B-cells) only through immunofluorescence and confocal microscopy, as the results obtained by immunoenzymatic techniques were unsatisfactory.

MZB-cells, like B1-cells, are part of the innate immune response and play essential roles in the early stages of humoral immune responses.^28^ MZB-cells reside in lymphoid follicles, particularly in the spleen, and efficiently transport antigens from the blood to the follicles, where they activate CD4+ T-cells and induce the differentiation of B-cells into plasma cells.^29^ In the present sample, MZB-1 expression was higher in tuberculoid leprosy than in the indeterminate form. Interestingly, MZB-cells expressing MZB-1 correlate with a higher number of infiltrating CD8+ T-lymphocytes in the tumor environment of pancreatic carcinoma and are associated with a better prognosis for this neoplasm.^20^

Analyzing the expression of CD20 and MZB-1 in the studied groups, the authors found that in the Tuberculoid (TT) leprosy group, CD20 expression was higher than that of MZB-1. This is probably because MZB-1 stains B1/MZB/plasma cells, and plasma cells are strongly associated with the Th2 (humoral) response in leprosy, which is scarce in the Th1 pole.^25^, ^26^ In addition, the authors observed that, in double-labeling techniques, MZB and B1-cells were much scarcer than CD20-positive cells in all groups of patients, including TT.

On the other hand, in the Lepromatous group (LL), CD20 expression was lower than that of MZB-1. In LL, the plasma cell component is significant, and in this context, the MZB-1 antibody would reveal plasma cells. The scarcity of double-stained B1 and MZB cells in LL and T2R samples supports the hypothesis that the MZB-1 antibody displayed mainly plasma cells rather than B-1/MZ B-cells.

In the type 2 reaction, the authors observed a higher expression of CD20 compared to MZB-1 expression. Given this finding, the authors can propose a similar hypothesis as in the case of the TT group: B1/MZB-cells were also limited in T2R, and existing literature does not indicate an abundance of plasma cells in T2R.^25^, ^26^

There was no significant difference in the indeterminate (I) leprosy group regarding the expression of the two markers, possibly due to the scarcity or absence of inflammatory cells.

There was also no difference in the expression of CD20 and MZB-1 in T1R. Considering that plasmablasts and plasma cells are recognized to be infrequent in T1R,^25^, ^26^ the few cells that exhibited MZB-1 immunostaining are likely MZB-cells or B1-cells. The most robust double-labeling for B1 and MZB-cells was observed in T1R lesions. In employing the PAX5/MZB-1 co-localization, the authors excluded plasmablasts and plasma cells, thereby restricting the labeling to B1/MZB-cells.

Interestingly, the disparity between the T1R, T2R, LL, I, and TT groups in PAX5/MZB-1 (B1 and MZB-cells) closely paralleled that observed in PAX5/CD5 (specifically marking B1-cells), suggesting that PAX5/MZB-1 did not display a substantial number of MZB-cells. For both markers (PAX5/MZB-1 and PAX5/CD5), T1R exhibited significantly more cells than T2R, LL, and I. However, uniquely in PAX5/MZB-1, the authors observed a significantly higher cell count in T1R than in TT. Since this discrepancy was not observed in the PAX5/CD5 labeling (B1-cells), the authors attribute it to a difference in the number of MZB-cells, which may be lower in the TT group.

While the authors did not observe a significant number of B1-cells in the lepromatous group, Kotb et al.,^30^ using peripheral blood flow cytometry, reported a higher percentage of these cells in lepromatous leprosy patients compared to healthy controls. They also associated the presence of B1-cells with autoimmune phenomena in the patient group.

The number of B-effector-1 cells (Be1) was higher in T1R and TT than in the I and T2R groups. The association of Be1-cells with a Th1 response makes this outcome expected. While not achieving statistical significance, LL showed a lower count of Be1-cells compared to TT and T1R, further reinforcing the connection of Be1-cells with the Th1 response in leprosy. Lund and colleagues demonstrated that Th1-cells, specifically those producing IFN-γ or stimulated by IL-12, induce the polarization of B-cells into Be1, leading to the expression of pro-inflammatory cytokines such as IFN-γ and IL-12.^15^ Conversely, interaction with Th2 cells or exposure to IL-4 directs B-cells, termed Be2, to produce IL-2 and IL-4. Be1 and Be2 cells, in turn, play roles in promoting the in vitro differentiation of Th1 and Th2 cells, respectively.^16^, ^31^ Subsequently, it was revealed that B-cell-derived IFN-γ plays a crucial role in Th1 responses, contributing to allograft and tumor rejection, autoimmune arthritis, and antibacterial response.^32^, ^33^, ^34^

Regulatory B-cells (Bregs) were notably scarce across all leprosy groups studied, with significant differences observed only between the T1R and I groups. It is important to note that, as mentioned earlier, MZB and B1-cells are subtypes of innate B-regulatory cells. However, the number of MZB and B1-cells was higher than that of Bregs. This apparent paradox may be attributed to the fact that the authors identified B regulatory cells based only on the expression of the IL-10 transcription factor c-MAF.

The predominance of Bregs in the Th1 pole aligns with prior studies that have highlighted the protective role of Bregs in various autoimmune diseases such as multiple sclerosis, systemic lupus erythematosus, rheumatoid arthritis, type 1 diabetes mellitus, and inflammatory bowel disease, all of which are associated with Th1 or Th17 cells.^35^ Additionally, Bregs have been implicated in tumor immunology exerting their immunosuppressive properties within the tumor microenvironment.^36^

Considering that this study, although it involved a large number of samples, is retrospective and relies on immunohistochemical techniques for identifying B lymphocyte subtypes, the authors have been able to demonstrate and compare the presence of these B-cell subsets across various leprosy lesion sites and reactional states. While there are limitations to this approach, these findings suggest distinct roles for these B lymphocytes, particularly within the tuberculoid pole and Type 1 leprosy reactions.

## Financial support

None declared.

## Authors' contributions

**Luis Alberto Ribeiro Fróes:** Conceptualization, Data curation, Formal analysis, Investigation, Methodology, Visualization, Writing - original draft. **Carla Pagliari:** Data curation, Investigation. **Maria Angela Bianconcini Trindade:** Data curation, Investigation, Methodology, Supervision, Writing - review & editing. **Mirian Nacagami Sotto:** Conceptualization, Data curation, Investigation, Funding acquisition, Methodology, Project administration, Supervision, Validation, Writing - review & editing.

## Conflicts of interest

None declared.
