# Supplementary material for: B-cell subsets in leprosy lesions: unraveling the complex interplay
Source: An Bras Dermatol. 2025 Aug 9;100(5):501184. doi: 10.1016/j.abd.2025.501184 (PMC12357089; doi:10.1016/j.abd.2025.501184)
Supplement: Supplementary file 1 [file mmc1.docx]

**ABD-D-24-00828**

**Supplementary Material**

**Methods**

Histological sections were obtained from paraffin-embedded material and mounted on charged slides for better adhesion. Subsequently, they were deparaffinized and hydrated. Endogenous peroxidase blocking was performed with hydrogen peroxide, followed by rinsing the slides in running water and distilled water. Antigen exposure was achieved using a retrieval solution (Dako), pH 9.0, for 20 minutes at 95ºC. Sections were again rinsed in running water, distilled water, and phosphate buffered saline (PBS), pH 7.4. The next step involved blocking nonspecific tissue proteins by incubation in a 10% skimmed milk solution (Molico, Nestlé) for 30 minutes at room temperature. The specimens were then incubated with primary antibodies, diluted in a 1% solution of bovine serum albumin (SERVA code 11930) supplemented with 0.1% sodium azide in PBS, pH 7.4, overnight at 4ºC. After two washes with PBS for five minutes each, incubation with the secondary anti-immunoglobulin antibody (rabbit or mouse) was carried out for 30 minutes at 37ºC. The specimens were again washed in PBS and incubated with the polymer systems specified in Table 1, consisting of two 30-minute incubation steps, composed of post-primary reagent and polymer. Following another wash in PBS, the reaction was revealed with a diaminobenzidine chromogenic solution (3,3'-diaminobenzidine, SIGMA Chemical Co., St. Louis, MO/USA, code D5637) 0.03%, supplemented with 1.2 ml of 3% hydrogen peroxide. The intensity of the brown color was microscopically controlled using positive controls accompanying each reaction. Histological sections were washed in running water for 10 minutes, counterstained with Harris hematoxylin for 10 seconds, washed in running water, dehydrated in ethanol, and clarified in xylene. The slides were mounted with resin.

**Immunohistochemical Techniques – Double Staining Reaction Protocol**

The first reaction was performed similarly to the protocol described above. However, 50 micrograms of nickel chloride were added to the diaminobenzidine solution, resulting in black staining. This protocol was used to visualize PAX-5 and Tbet.

Subsequently, the specimens were rinsed in running water, distilled water, and PBS buffer, and the second immunohistochemical reaction was performed, with incubation of the second primary antibody and revelation of the reaction also with polymer-based systems. Positivity was visualized with the "Permanent-green" kit, Bio-SB, for the detection of CD5, IL10, and CD20, concomitant with PAX-5 and Tbet.

**Morphometric and Statistical Analyses**

The images were analyzed using the free Fiji / ImageJ software from NIH ([LTtp://imagej.nih.gov](http://imagej.nih.gov/)). After calibration, each image was converted into an RGB-color image, turning immunostained areas red. Immunostained areas were converted to red, and the positive area was measured. Finally, the dermal area of the image was stained red, and measured. The positively stained area was then divided by the total dermal area in square micrometers. After analyzing all images for each sample, the arithmetic mean of the immunostained area fraction (CD20 and MZB-1) was calculated for each sample (case).

|  | **CD20** | **MZB1** | ***p*-value*** |
| --- | --- | --- | --- |
| **TT** | 0.00981 | 0.00312 | 0.0000045 |
| **I** | 0.00002 | 0.00000 | NS |
| **LL** | 0.00019 | 0.00361 | 0.000465 |
| **T2R** | 0.00596 | 0.00127 | 0.007633 |
| **T1R** | 0.00208 | 0.00225 | NS |

**Table 1S** This table compares the median fractions of immunomarked areas for CD20+ and MZB-1+ cells across different groups. TT, tuberculoid leprosy; I, indeterminate leprosy; LL, lepromatous leprosy; T2R, Type 2 reaction; T1R,Type 1 reaction; NS, Non-statistically significant. *Mann-Whitney test.
